# Editorial: Reading and writing skills: cognitive, emotional, creative, and digital approaches

**DOI:** 10.3389/fpsyg.2023.1279276

**Published:** 2023-11-27

**Authors:** María Isabel de Vicente-Yagüe Jara, Pedro García Guirao, Elena del Pilar Jiménez-Pérez, Olivia López Martínez

**Affiliations:** ^1^Department of Didactics of Language and Literature, University of Murcia, Murcia, Spain; ^2^Department of Linguistics, WSB University in Wrocław, Wrocław, Poland; ^3^Department of Didactics of Language, Art and Sports, University of Málaga, Málaga, Spain; ^4^Department of Didactics of Evolutionary and Educational Psychology, University of Murcia, Murcia, Spain

**Keywords:** reading, writing, cognitive, emotional, creative, digital

Within the Research Topic “*Reading and Writing Skills: Cognitive, Emotional, Creative, and Digital Approaches*”, 40 articles were published in the “Educational Psychology” section of the following two journals in a continuous publication: *Frontiers in Psychology* and *Frontiers in Education*. These are studies conducted in an educational context by researchers from Italy, Portugal, Spain, the Netherlands, Poland, Sweden, the United Kingdom, the United States, Canada, Peru, Mexico, Colombia, Turkey, Iran, China, Saudi Arabia, Pakistan, Malaysia, and New Zealand.

The development of literacy, defined as the psycholinguistic and social components of communication, is crucial in the educational context from primary school to university. Although reading and writing are two well-studied pillars of education, this Research Topic is currently interested in rethinking these educational processes, highlighting the cognitive, emotional, creative, and digital needs that have emerged as a result of the pandemic, using a multidisciplinary and holistic approach.

With confinement limits and social distancing measures, we are now trying to operate flexibly in a new health situation resulting from the worldwide outbreak of COVID-19. The growing “new normal” has reshaped instructors' roles, techniques, and work schedules. Similarly, increasing digital literacy and developing strategies for mastering new skills are critical components of using, interpreting, communicating, and sharing information in this environment. In addition, parental involvement in the student learning process impacts not only academic success, but also a variety of emotional and social issues. In addition to the changing role of teachers and the affective social impact of the family, literacy skills can be useful in nurturing creativity.

Within this thematic perspective, the published articles have addressed the study of literacy from a variety of perspectives that provide an in-depth look at the current diverse educational landscape, as described below.

First, both reading comprehension and writing require the interaction of several cognitive processes. If we start with the learning of these skills at an early age, we find the studies “*A longitudinal study on sensitivity to symmetry in writing and associations with early literacy abilities*” (Yin and McBride); and “*A study on the emergence of sound-sign correspondence in Italian-speaking 5-year-old pre-schoolers*” (Bigozzi et al.).

Following the cognitive strategies involved in reading skills, these are addressed in relation to morphological awareness, grammatical knowledge, text genres, eye movements, school belonging, academic achievement between linguistic and non-linguistic subjects. This is the case of the articles entitled “*Longitudinal contributions of morphological awareness, listening comprehension, and gains in word reading fluency to later word- and text-reading fluency*” (Metsala); “*The relationship between grammatical knowledge and reading comprehension: A meta-analysis*” (Zheng et al.); “*Cognitive Strategies and Textual Genres in the Teaching and Evaluation of Advanced Reading Comprehension (ARC)*” (García-Sánchez and García-Martín); “*Eye movements are stable predictors of word reading ability in young readers*” (Strandberg et al.); “*School Belonging and Reading Literacy: A Multilevel Moderated Mediation Model*” (Tan et al.); “*Relationships among students' reading habits, study skills, and academic achievement in English at the secondary level*” (Abid et al.); and “*Influence of performance in Spanish language and literature on physical education and music grades*” (Clares-Clares and Gómez-Mármol).

In line with the articles on reading comprehension, the study “*Vocabulary Repetition Following Multisensory Instruction Is Ineffective on L2 Sentence Comprehension: Evidence from the N400*” (Pishghadam et al.), which used electroencephalography to record students' electrophysiological neural activity, is also noteworthy.

Within this general background, learning difficulties in reading are studied from both prevention and intervention perspectives in “*The Importance of Phonological Awareness in Learning Disabilities' Prevention: Perspectives of Pre-School and Primary Teachers*” (Veríssimo et al.); and “*An Intervention in Reading Disabilities Using a Digital Tool During the COVID-19 Pandemic*” (Cadime et al.).

Continuing with the teaching and learning of writing, the relevance of writing strategies in the cognitive processes of expression is studied in the articles “*Validation of the Writing Strategies Questionnaire in the Context of Primary Education: A Multidimensional Measurement Model*” (Arias-Gundín et al.); and “*What spelling errors can tell us about the development of processes involved in children's spelling*” (Niolaki et al.).

In addition, the specific case of learning Chinese characters is addressed in the study “*Comparing the Effects of Stroke-Appearing and Stroke-Disappearing on Learning the Order of Strokes in Chinese Characters*” (Hong et al.).; and “*Reliability, validity, and measurement invariance of a Chinese handwriting legibility scale among primary students in central China*” (Lu et al.).

Among the cognitive processes involved in writing, the mental operations of reasoning, interpreting, and arguing are of paramount importance in academic writing, as discussed in articles such as “*Analysis of pre-service teachers' argumentation-based academic writing process*” (Direkci et al.); “*Investigating Effects of Small-Group Student Talk on the Quality of Argument in Chinese Tertiary English as a Foreign Language Learners' Argumentative Writing*” (Li and Zhang); “*Implicit Teacher Theories Regarding the Argumentative Commentary of Multimodal Texts in the Teaching of Spanish as a Native and Foreign Language*” (Caro Valverde et al.); and “*Effects of cognitive task complexity and online planning on second language learners' argumentative writing*” (Xu and Zhang).

In particular, the belief in one's own ability to communicate successfully through writing has been studied from the perspective of self-efficacy in several published articles, as is the cases of “*Psychometric properties and invariance of the self-efficacy for writing scale in Peruvian high school students”* (León-Gutiérrez et al.); and “*Development and validation of a genre-based second language (L2) writing self-efficacy scale*” (Zhang et al.). Moreover, self-efficacy can regulate anxiety and other emotional variables, as seen in the articles “*Research on correlation between English writing self-efficacy and psychological anxiety of college students*” (Li); and “*Investigating high schoolers' L2 writing anxiety, L2 writing self-efficacy, L2 writing self-regulated strategies, and L2 writing engagement: Relationships and mediator*” (Zhou et al.).

Second, regarding the emotional education approach in language and literature teaching and learning processes, recent and significant publications on this Research Topic stand out. In recent years, a great deal of research has been published on reading literacy. However, there are hardly any studies that link reading literacy with emotional intelligence. In this line we find the article “*Emotions and reading: When reading is the best way to improve skills in adolescents*” (Jiménez-Pérez et al.), in which evidence was obtained of a direct relationship between both variables, especially in the experimental group in which the reading habit was stimulated.

From a learner resilience perspective, the article “*The role of learner character strengths and classroom emotions in L2 resilience*” (Alrabai and Alamer) examines a theory-based model to explain how trait emotional intelligence and language learner effort, as two-character strengths, predict learner enjoyment (positive emotions) and anxiety and boredom (negative emotions), and how these variables together predict resilience in language learning.

Moreover, motivation in the reading process is one of the main concerns of teachers today, as can be seen in the articles “*Beyond the Educational Context: Relevance of Intrinsic Reading Motivation During COVID-19 Confinement in Spain*” (De Sixte et al.); and “*By Toutatis! Trainee Teachers' Motivation when Using Comics to Teach History*” (Moreno-Vera et al.).

Finally, the topic of the affective social influence of the family on the learning of reading is addressed in the following article: “*The integration of affective characteristics of the family environment for a more comprehensive explanatory model of reading abilities*” (Gagné et al.).

Thirdly, we find the role that creativity is playing in recent years in the educational field, in general, and in language learning and writing practice, in particular. This is the case of certain studies published in this Research Topic, which connect student creativity with digital practices, such as “*The Role of Digital Technologies to Promote Collaborative Creativity in Language Education*” (Selfa-Sastre et al.); and “*From Text on Paper to Digital Poetry: Creativity and Digital Literary Reading Practices in Initial Teacher Education*” (Selfa-Sastre and Falguera Garcia).

Creative expression is addressed in another study that examines narrative textual competence, both oral and written, entitled “*Contribution of oral narrative textual competence and spelling skills to written narrative textual competence in bilingual language-minority children and monolingual peers*” (Vettori et al.).

On the other hand, the creative development of students is also studied based on the image as language, in an article that aims to delve into the visual message in its productive and interpretive aspects, entitled “*The Image as Language: The Creation and the Use of the Visual Message by Young University Students in Their Communicative Social Activity*” (Ramón-Verdú and Villalba-Gómez).

Finally, in relation to the predominance of the digital era in the educational context, which requires the development of new skills and learning models, the article entitled “*Digital literacy in the university setting: A literature review of empirical studies between 2010 and 2021*” (Gutiérrez-Ángel et al.) aims to analyze the empirical evidence provided by international research related to digital literacy among university students.

More specifically, the use of information and communication technologies for language learning has been particularly studied in the contexts of Portugal and China, through the articles “*Predictors of Portuguese teachers' use of Information and Communication Technologies in literacy classes*” (Nunes et al.); and “*Internet use predicts Chinese character spelling performance of junior high school students: multiple mediating roles of pinyin input proficiency and net-speak experience*” (Luo et al.). Similarly, the pedagogical relevance of using artificial intelligence for learning English as an L2 is addressed in the article “*Research on Papua, a digital tool with artificial intelligence in favor of learning and linguistic attitudes toward the learning of the English language in students of Spanish language as L1*” (Peña-Acuña and Crismán-Pérez).

Likewise, literature is not excluded from this digital universe, which is reflected in the promotion of children's picturebooks in the study “*The promotion of critical reading through the digital environment: A study on the virtual epitexts used to promote children's picture books*” (Tabernero-Sala and Colón-Castillo). Similarly, reading-literary cooperation through spaces of participatory culture is developed in the article “*Mechanisms for Interpretative Cooperation: Fan Theories in Virtual Communities*” (de Amo and García-Roca).

Finally, the article “*Academic literacy among university students in Mexico and Spain: A holistic perspective*” (Castillo-Martínez et al.) aims to identify the extent to which cognitive, emotional, attitudinal, digital and personality aspects influence the development of academic literacy among university students.

In conclusion, through the reading of the different articles that make up this Research Topic, the value of a holistic view that integrates the study of literacy from a pluralistic research perspective has been demonstrated in order to meaningfully delve into the educational reality and be able to face the current problems, challenges and opportunities presented by the teaching and learning of reading and writing.

## Author contributions

MV-Y: Conceptualization, Writing—original draft, Writing—review & editing. EJ-P: Writing—review & editing. OL: Writing—review & editing. PG: Writing—review & editing, Writing—original draft.

